# Human Ribosomal RNA-Derived Resident MicroRNAs as the Transmitter of Information upon the Cytoplasmic Cancer Stress

**DOI:** 10.1155/2016/7562085

**Published:** 2016-07-19

**Authors:** Masaru Yoshikawa, Yoichi Robertus Fujii

**Affiliations:** ^1^Pharmaco-MicroRNA Genomics, Graduate School of Pharmaceutical Sciences, Advanced Pharmaceutical Science Center, Nagoya City University, Nagoya 467-8603, Japan; ^2^Retroviral Genetics Group, Graduate School of Pharmaceutical Sciences, Advanced Pharmaceutical Science Center, Nagoya City University, Nagoya 467-8603, Japan

## Abstract

Dysfunction of ribosome biogenesis induces divergent ribosome-related diseases including ribosomopathy and occasionally results in carcinogenesis. Although many defects in ribosome-related genes have been investigated, little is known about contribution of ribosomal RNA (rRNA) in ribosome-related disorders. Meanwhile, microRNA (miRNA), an important regulator of gene expression, is derived from both coding and noncoding region of the genome and is implicated in various diseases. Therefore, we performed* in silico* analyses using M-fold, TargetScan, GeneCoDia3, and so forth to investigate RNA relationships between rRNA and miRNA against cellular stresses. We have previously shown that miRNA synergism is significantly correlated with disease and the miRNA package is implicated in memory for diseases; therefore, quantum Dynamic Nexus Score (DNS) was also calculated using MESer program. As a result, seventeen RNA sequences identical with known miRNAs were detected in the human rRNA and termed as rRNA-hosted miRNA analogs (rmiRNAs). Eleven of them were predicted to form stem-loop structures as pre-miRNAs, and especially one stem-loop was completely identical with* hsa-pre-miR-3678* located in the non-rDNA region. Thus, these rmiRNAs showed significantly high DNS values, participation in regulation of cancer-related pathways, and interaction with nucleolar RNAs, suggesting that rmiRNAs may be stress-responsible resident miRNAs which transmit stress-tuning information in multiple levels.

## 1. Introduction

It has recently been revealed that dysregulation of ribosome biogenesis is implicated in various diseases termed ribosomopathy such as Diamond-Blackfan anemia (DBA), Shwachman-Diamond syndrome (SDS), X-linked dyskeratosis congenita (DKC), Treacher Collins syndrome (TCS), and cartilage hair hypoplasia (CHH) [1–3]. The most studied ribosomopathy, DBA, is a rare congenital hypoplastic anemia and its pathogenesis is associated with defects in various ribosomal protein (RP) genes such as RPS19, RPS24, RPL5, and RPL11. Mutation in RPS and RPL genes results in significant reduction in the amount of mature 40S and 60S subunit, respectively [4]. Other ribosomopathies, SDS, DKC, TCS, and CHH, are caused by gene defects on SBDS, DKC1, TCOF1, and RMRP, respectively, which encode proteins involved in ribosome biogenesis [2]. However, what mechanism is linked to these proteins in the pathogenesis of ribosomopathies? Whether cancer is related to them? These are still unsolved.

Ribosomal RNA (rRNA) is the most abundant noncoding RNA gene in cells and is essential for the structure and function of ribosomes. All four eukaryotic rRNAs, such as 18S, 5.8S, 28S, and 5S, are highly conserved across human and related species, and their biogenesis is strictly regulated by several mechanisms [5–8]. RNA45S, also called RN45S, the 45S gene, or rDNA, is an operon containing 18S, 5.8S, and 28S RNA genes [8–11]. On the other hand, the 5S RNA gene is coded alone. Among eukaryotes, the RNA45S and 5S RNA genes are transcribed by Pol I and Pol III, respectively [12, 13]. The first step in rRNA gene transcription in humans is the formation of the preinitiation complex (PIC) on the core promoter and the upstream control element of rDNA. PIC attracts Pol I, and a full-length rRNA precursor called 47S rRNA is transcribed. 47S rRNA is processed into 45S rRNA by cleaving fixed positions on the 3′ and 5′ external transcribed spacers (ETS) in the nucleus and is then divided into 21S and 32S rRNA by either of the two processes [8]. Finally, 18S-E, 6S, and 28S rRNAs are generated through various mechanisms and transported into the cytoplasm to construct the mature ribosomal complex.

RNA45S genes in humans are located on chromosomes (Chr) 13, 14, 15, 21, and 22 [14]. These acrocentric chromosomes have multiple copies of the 45S RNA gene on the p12 region in their short arms. This tandemly repeated rRNA gene copy is commonly called an rDNA repeat or rRNA gene cluster, and each repeating unit consists of a nontranscribed spacer (NTS) and the RNA45S gene. RNA45S also contains a 5′ ETS, an internal transcribed spacer (ITS), and a 3′ ETS in addition to the 18S, 5.8S, and 28S rRNA genes. On the other hand, the 5S rRNA gene is only located at the q42 region of chromosome 1. The copy number of rDNA is important for normal cell functions although the majority of rDNA copies are transcriptionally silent; therefore, reduced rDNA copy number after cell stress is repaired by a specific amplification system. It has also been reported that perturbation in the copy number and stability of rRNA gene caused by mutations in rRNA-related enzymes or cell senescence are linked to various cellular dysfunctions and insufficiency of genome integrity [15–18].

MicroRNA (miRNA) is an essential regulator of gene expression and a member of the small noncoding RNA family, which are RNAs approximately 22 nucleotides long [19]. Sequence complementarity-based interactions between miRNA and its target mRNA suppress and occasionally augment the translation of mRNAs into proteins [20–23]. One miRNA regulates multiple mRNAs; thus, one mRNA is targeted by multiple miRNAs [24–26]. Almost all functional genes in humans are under the control of miRNAs [27]. Therefore, alterations in the miRNA profile after injury, infection, or chemical treatment can alter various functions, such as immunoreactivity, cell proliferation and differentiation, apoptosis, and carcinogenesis [28–32]. The expression profile of miRNA genes is deeply associated with a considerable number of human diseases including cancer [28, 29]. There are several reports about miRNA dynamics after cell stresses, for instance, participation of poly(ADP-ribose) in controlling miRNA activity in the cytoplasm [33]. In the deep insight of miRNA-disease relationship, it needs huge efforts to make complete data for clinical validation of miRNA-mRNA associations in diseases. Therefore, it has been shown that computational analysis is required for miRNA research and increasing number of disease-related miRNA databases and computational analyses have recently been established [34, 35]. The miRNA genes are scattered throughout the genome, and miRNAs are created through many complexed processing pathways [36–38]. Most miRNA genes are transcribed by RNA polymerase II (Pol II) as hairpin-shaped primary miRNA (pri-miRNA), and the pri-miRNA is processed into pre-miRNA after cleavage of the 5′-cap and 3′-polyA tail by the microprocessor complex, which is composed of Drosha and DiGeorge syndrome chromosomal region 8 (DCGR8). These are the RNase III proteins and double-stranded RNA binding proteins, respectively. Subsequently, pre-miRNA is exported to the cytoplasm by exportin-5 and further processed into the miRNA: miRNA^*∗*^ duplex by cleavage of the 5′- and 3′-termini and loop domain by Dicer, which is an RNase III-like protein. This duplex is finally loaded into the RNA-induced silencing complex, and a duplex chain is selected thermodynamically to function as mature miRNA [39]. However, some noncanonical pathways are used to mature miRNA [36, 40]. For example, dme-mir-1003 is the first discovered mirtron, which is a pri-miRNA that exists as an intron of pre-mRNA and is processed into pre-miRNA without the Drosha canonical processor [41]. This means that all protein-coding, noncoding, intergenic, and intragenic regions can become miRNA hosts.

According to the RNA wave 2000 model advocated by Fujii, miRNA genes are the RNA information genes with four critical characteristics: (1) the miRNA gene is a mobile genetic element that induces transcriptional and posttranscriptional silencing via networking processes; (2) the RNA information supplied by miRNA genes expands to intracellular, intercellular, intraorgan, interorgan, intraspecies, and interspecies under a lifecycle in the global environment; (3) mobile miRNAs self-proliferate; and (4) cells contain resident and genomic miRNAs [42, 43]. miRNAs can be classified into genomic and resident miRNAs. The former are miRNAs preserved in DNA as miRNA genes, and the latter are miRNAs stored in a non-DNA form. The greatest difference between genomic and resident miRNAs is the expression regulatory mechanism. Most known miRNAs are genomic because their expression levels are controlled by a specific transcriptional factor, RNA polymerase, and so forth [44–46]. However, some miRNAs, such as mmu-miR-712, dme-miR-10404, and hsa-miR-663, are typical resident miRNAs because they do not require specific transcriptional factors or nucleases to exert their functions [47, 48].

In particular, it is anticipated that resident miRNAs and other cytoplasmic RNAs play more important roles in cells with unique cytoplasmic or genomic characteristics, such as erythrocytes, spermatozoa, and oocytes, than those of other cells. Erythrocytes contain diverse and abundant RNA species, including cytoplasmic miRNAs that contribute to regulating erythropoiesis and malarial resistance, although erythrocytes have been thought to contain no RNA because they are anucleated [49, 50]. Given that erythrocytes are the most abundant cell in blood, a large number of erythrocyte-contained miRNAs may be circulating. Spermatozoa are characterized by minimal cytoplasm and extremely condensed DNA. However, various RNAs are abundant in the cytoplasm of spermatozoa, such as rRNA, transfer RNA (tRNA), piwi-interacting RNA, and miRNA, and have important roles before and after fertilization [51–53]. Oocytes are transcriptionally silent cells; therefore, the many pooled mRNA and noncoding RNAs in the cytoplasm, such as miRNAs, are essential to complete late oogenesis and early embryogenesis without de novo transcription [53, 54]. Only the resident RNAs in these cells are considered information transmitters or memorizing devices, rather than DNA. Furthermore, as miRNA is self-reproducible, an identical miRNA could become both genomic and resident miRNA [42]. The quantities of tRNA and rRNA decrease under stress, suggesting that resident miRNAs help with biological regulation under stress [55]. Thus, we hypothesized that cytoplasmic tRNA and/or rRNA is a pool of self-reproducible resident miRNAs.

tRNA is another functional noncoding RNA that is most abundant (approximately 10% of RNAs) in cells next to rRNA (approximately 80% of RNAs). Recent studies have discovered that transfer RNA-derived RNA fragments (tRFs) are generated from tRNAs as terminal functional products. In the case of murine gammaherpesvirus 68, viral miRNAs were generated by Pol III [56]. Further, a number of endoribonucleases including Dicer and Angiogenin are implicated in the production of tRFs from tRNA transcripts [57, 58]. tRFs exist in various species, such as humans, cows, flies, and plants, and work as gene expression regulators, similar to miRNA [57, 59–63]. Some tRFs were listed as miRNAs in the miRBase (now dead entries). Other common characteristics between miRNAs and tRFs are their interactions with Argonaute (AGO) proteins, significant changes in expression levels during disease and aging, and circulation in a steady form [61–63]. Several reports have shown that tRFs are occasionally more abundant than miRNAs [64].

We considered the possibility that RNA fragments may be derived from rRNA in a manner similar to how tRFs are derived from tRNA because several tRF-related endoribonucleases have common activity of nuclease [58]. Till date, to the best of our knowledge, only a few studies have reported biogenesis and functions of rRNA-derived miRNAs or miRNA-like fragments, although many rRNA-annotated fragments of miRNA-like size have been detected in deep sequencing data from RNA studies [65]. Chak et al. revealed that the novel miRNA hairpin named mir-10404/mir-ITS1 exists in the ITS1 region of* Drosophila* rDNA [47]. Son et al. also discovered that mmu-miR-712 is coded in ITS2 of mouse 45S precursor RNA (Rn45s) and hsa-miR-663 is coded in the ITS1 region of human RNA45S [48]. Furthermore, Drosha-related proteins are included in rRNA processing pathways [66]. These ITS-derived miRNAs are supposed to be generated upon degradation of the ITS region, similar to the generation of mirtrons in the nucleoplasm or cytoplasm.

The effects of tRNA or rRNA degradation and processing on cell activities in response to stress are important. The small RNA molecules derived through this process play an important role in the transition from fine-tuning to stress-tuning functions. Other ncRNA species such as SINE, especially human Alu elements, have also been revealed to be contained in nucleolus and control the size of nucleoli adopting to cell circumstances [67].

Therefore, we examined whether rRNAs contain functional small RNAs and confirmed the relationship between ribosome and disease shown in previous studies. Moreover, how rRNA-hosted microRNA analogs (rmiRNAs) contribute to the stress response as nongenomic memory in the nucleolus and cytoplasm was also investigated using multiple computer-based tools and databases to find stress-tuning RNA interaction in transcriptional and posttranscriptional level. The quantum relationships among miRNAs were also calculated as Dynamic Nexus Score (DNS) by MESer program that we have previously developed and its significance in stress response was discussed.

## 2. Method

### 2.1. Sequence Data Collection

All miRNA sequence data used in this study were downloaded as miRNA.dat, hairpin.fa, and mature.fm from miRBase (http://www.mirbase.org) in release 21 (June 2014) [68]. This includes 2,588 and 1,915 mature miRNA sequences of human and mouse, respectively. Sequences of rRNAs were obtained from European Nucleotide Archive (ENA, http://www.ebi.ac.uk/ena) release 127 (April 6, 2016) and National Center for Biotechnology Information (NCBI, http://www.ncbi.nlm.nih.gov) in FASTA format [69]. After comparing and merging latest rRNA sequence data, two rRNA coding sequences, RNA45S and human rDNA complete repeating unit, were selected as the source of rRNA sequence (Supplemental Table  1 in Supplementary Material available online at http://dx.doi.org/10.1155/2016/7562085). The latter source involves the sequence of the former but their sequences have some differences even in common regions, for instance, slightly polymorphisms in 18S and moderate ones in 28S, ITS, and ETS region. Sequences of tRNA and tRF were also obtained from Genomic tRNA database (GtRNAdb, http://gtrnadb.ucsc.edu) and tRFdb (http://genome.bioch.virginia.edu/trfdb), respectively [70, 71].

### 2.2. Definition of Passenger Strand

Passenger strands of miRNAs whose guide strands were found in the rRNA sequences were researched referring to stem-loop structure in miRBase. If a passenger strand is not recorded in miRBase, a sequence which is complement to the guide strand was defined as the passenger strand in this study.

### 2.3. Secondary Structure Prediction

To determine the secondary structures of found miRNA-like sequences, M-fold was used in a condition of 37 Celsius degrees and 1 M NaCl. Any other options which influence prediction results were set in default (RNA sequence is linear, percent suboptimality number is 5, upper bound on the number of computed foldings is 50, the window parameter is default, the maximum interior/bulge loop size is 30, the maximum asymmetry of an interior/bulge loop is 30, and the maximum distance between paired bases is no limit).

### 2.4. Chromosome Confirmation

For browsing miRNA locations on each chromosome visually, UCSC Genome Browser on Human Dec. 2013 (GRCh38/hg38) Assembly (https://genome.ucsc.edu/cgi-bin/hgTracks?db=hg38) was used.

### 2.5. Calculations of DNS

Dynamic Nexus Score (DNS) was prepared as a quantum-based score for evaluating quantum interactions between or among miRNAs [72]. DNS calculation of rRNA-derived miRNA and tRF was performed by using the original program, MESer (http://meser.mirna-academy.org). Computational results were statistically analyzed with Microsoft Office Excel 2013 (Microsoft Japan Co., Ltd., Tokyo, Japan).

### 2.6. Target Prediction and Ontology Analysis

Putative targets of rmiRNAs were predicted under the seed theory by using TargetScan (http://www.targetscan.org/) [73]. Validated targets of rmiRNAs were confirmed in miRTarBase (http://mirtarbase.mbc.nctu.edu.tw/) [74]. Selection of top 10 targets in miRTarBase was conducted by referring to the number of validation methods (primary) and the number of reports (secondary). Categorization of putative target genes in Gene ontology (GO) and Kyoto Encyclopedia of Genes and Genomes (KEGG) pathways was accomplished by using GeneCoDis3 web service (http://genecodis.cnb.csic.es/) [75].

### 2.7. Alu Sequence and Target Site Prediction

Sequence data of human Alu family were downloaded from SINE Base (http://sines.eimb.ru), last update May 28, 2015 [76]. Target sites of rmiRNAs in Alu sequences were predicted using RNA22 version 2.0 (https://cm.jefferson.edu/rna22/) with default settings (sensitivity of 63%, specificity of 61%, seed size of 7, allow maximum of 1 UN-paired bases in seed, minimum number of paired-up bases in heteroduplex being 12, maximum folding energy for heteroduplex being −12 Kcal/mol, and maximum number of G : U wobbles allowed in seed region being no limit) [77].

## 3. Results

### 3.1. Pre-miRNA Sequence in Human rRNA

To investigate whether miRNAs also exist in human rRNA, we firstly collected base sequences of pre-miRNAs, mature miRNAs, and rDNA. Then the sequence of rRNA and its adjacent regions, RNA45S, and rDNA-repeating unit, respectively, were searched for 2,588 human pre-miRNA sequences by using a simple C++ based detection program we developed for this study. As a result, an identical sequence to pre-miR-3687 was detected from rDNA-repeating unit although the known location of the miR-3687 gene was distinct from rRNA coding region. However, other 2,587 pre-miRNA sequences were not found in any rRNA-related sequences. For further similar sequencing research, a detection of mature miRNA sequences instead of pre-miRNA from rRNA gene was also performed. Subsequently, seventeen RNA alignments identical to human mature miRNAs, namely, miR-663a, miR-663b-3p, miR-1268a, miR-1268b, miR-1275, miR-3648, miR-3656-3p, miR-3687-3p, miR-4417, miR-4466, miR-4488, miR-4492-3p, miR-4508, miR-4516, miR-4532, miR-6087, and miR-6724, were detected from the human rRNA sequences (Table 1). In detail, miR-1268a and miR-1268b were detected from only RNA45S, and miR-1275, miR-3687, and miR-6724 were detected from only rDNA-repeating unit.

Among these detected miRNAs, miR-1268a, miR-3648, miR-3687, miR-4508, and miR-6724 were originated in rDNA containing chromosomes, Chr 15, Chr 21, Chr 21, Chr 15, and Chr 21, respectively. However, their locations were different from rRNA coding regions (data not shown). This suggests that the detected miRNAs might also have been transcribed from rRNA-related regions as well as above Chr loci or that the miRNAs may be generated through further processing of transcribed rRNA gene.

**Figure 1:**
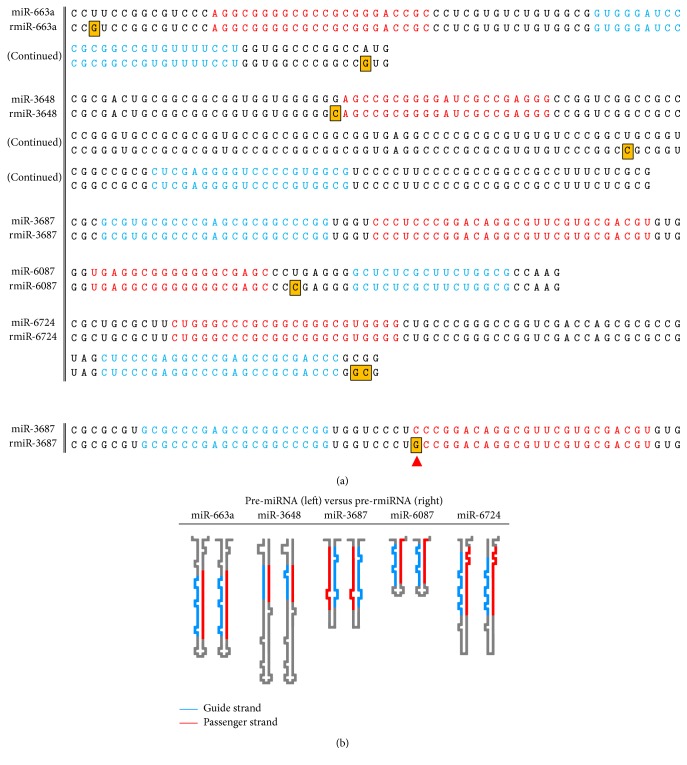
An overview of pre-rmiRNAs with high similarity to canonical pre-miRNAs. (a) Sequence comparison between detected precursor-rmiRNAs (pre-rmiRNAs) and their canonical pre-miRNAs. Mature (guide) miRNA strand is colored with red and passenger is colored with blue. Differences in base sequences between pre-rmiRNAs and pre-miRNAs are highlighted in yellow and box lines. (b) Comparison of secondary structure of pre-rmiRNAs with that of their canonical pre-miRNAs. These rmiRNAs contain the identical sequences to canonical ones in both guide and passenger. This group contains only a few polymorphisms in loop and terminal region.

To further examine whether the detected miRNAs could form miRNA/miRNA^*∗*^ duplex and/or stem-loop structure like miR-3687, their passenger strand sequences were also searched in the rDNA repeat region. Passenger strand sequences were determined referring to their putative stem-loop structure in miRBase. As a result, passenger strands of miR-663a, miR-3648, miR-3687, miR-6087, and miR-6724 were found nearby their guide strands (Figure 1(a)). This result indicated that these rRNA-hosted miRNA-like RNAs could form stem-loop or at least miRNA/miRNA^*∗*^ duplex. Intriguingly, a passenger strand of miR-3687 was detected from RNA45S although its guide strand was not detected. Since these rRNA-hosted RNA pieces, especially miR-3687, possess high concordance rate to each known pre-miRNA sequence, therefore we termed them rRNA-hosted miRNA analog (rmiRNA).

For more rigorous verification, putative precursor sequences of rmiRNAs were predicted by referring to the sequences and structures of known human pre-miRNAs identical to detected rmiRNAs (Figure 1(a)). Subsequently, secondary structures of pre-rmiRNA sequences were predicted by using M-fold software. The same prediction for canonical pre-miRNA sequences were also performed and used as positive controls for comparison, and it was proven that all of five pre-rmiRNA candidates could form hairpin-loop structures which have high similarities to that original pre-miRNAs form (Figure 1(b)).

### 3.2. Exploration for Noncanonical Passenger Strands and Precursors

Since one pre-rmiRNA sequence is identical with pre-miR-3687 and four pre-rmiRNAs which have high similarity to known pre-miRNAs were detected in rDNA, we thought that other twelve rmiRNAs also could form stem-loop structure with different style. To examine this hypothesis, we have carefully investigated adjacent regions of detected guide strands. Primarily, some RNA sequences were clipped out as putative pre-rmiRNA. Each of them contained guide strand sequence and had the same length to its canonical pre-miRNA. Next, these putative pre-rmiRNAs were compared with its canonical pre-miRNA in base sequences and then secondary structures. Of twelve putative pre-rmiRs, pre-rmiR-663a showed the highest similarity to pre-miR-663b in both base sequence and precursor structure (Supplemental Figure  1A). Other three pre-rmiRNAs, pre-rmiR-3656, pre-rmiR-4417, pre-rmiR-4466, and pre-rmiR-4508 also showed high similarity in secondary structures to pre-miR-3656, pre-miR-4417, pre-miR-4466, and pre-miR-4508, respectively, although their precursor sequences showed low similarities to canonical ones (Supplemental Figure  2A). These results implied that these rmiRNAs have obtained new passenger strand to maintain their function as mature miRNAs. On the other hand, putative pre-rmiR-1268b sequence generated in accordance with the rules above did not form stem-loop structure according to M-fold prediction. However, we found that pre-rmiRNA-1268b could construct stem-loop structure with a slight modification such as lengthening of the 3′ terminal region (Supplemental Figure  1A).

Furthermore, it was ascertained that the left six rmiRNAs, namely, rmiR-1268a, rmiR-1275, rmiR-4488, rmiR-4492, rmiR-4516, and miR-4532, also could form stem-loop structure by further modification. We conceived an idea of “reversed pattern” of primary structure; for instance, miR-1275 usually exists as 5p sequence in pre-miR-1275 but might exist as 3p sequence in pre-rmiR-1275. To examine this idea, broader region analysis was performed and some new candidate rmiRNA sequences were predicted (Supplemental Figure  1B). As a result, it was confirmed that all of new rmiRNAs can form well-ordered stem-loop structure (Table 2 and Supplemental Figure  2B).

### 3.3. DNS Computation and Comparison

Because concordance of so many sequences must not be detected accidentally, it is natural to consider hidden mechanisms on the background. We previously developed a quantum-based score, Dynamic Nexus Score (DNS), to evaluate miRNA/miRNA interactions and demonstrated that biological activity of the miRNA synergy is positively correlated with DNS value. The average DNS value among mature rmiRNAs was calculated through MESer computer program. DNSs of 1,032 human tRFs and all of 2,588 human miRNAs were also calculated as controls. Surprisingly, the average DNS of rmiRNAs marked 130.23; it was much higher than that of tRFs (40.76) and all human miRNAs (38.31) (Supplemental Figures  3A and 3B). Additionally, DNS values between tRFs, rmiRNAs, and all miRNAs were also calculated. As a result, it was confirmed that the miRNA pairs including rmiRNAs had relatively high DNS values (Supplemental Figure  3C), and this meant that rmiRNAs might induce miRNA-miRNA synergy to accelerate their biofunctions.

### 3.4. Target Prediction and Ontology Analysis

**Figure 2:**
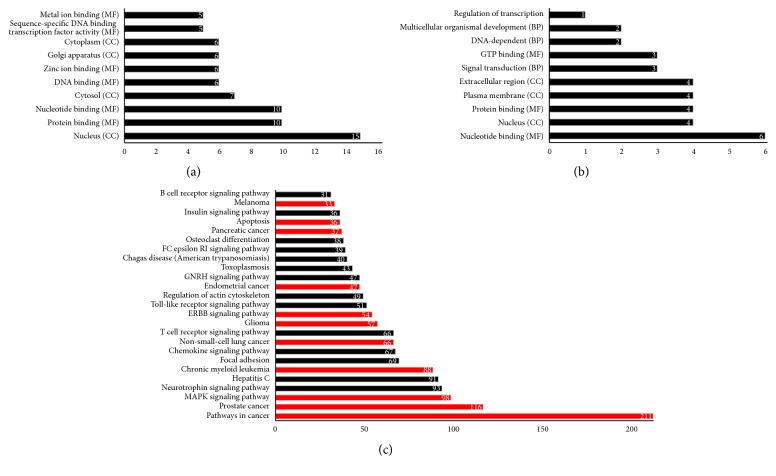
GO and KEGG analysis for predicted targets of rmiRNAs. (a) GO characterization of top 10 targets of the top 5 high DNS rmiRNAs in miRTarBase. (b) GO characterization of top 10 putative targets of the top 5 high DNS rmiRNAs in TargetScan. (c) KEGG pathway annotations of putative target genes having less than −0.1 cumulative weighted context++ score in TargetScan. Cancer and cancer-related pathways were colored with red.

To investigate targets of rmiRNAs, we used TargetScan and miRTarBase. TargetScan was used for collecting putative target genes predicted by the seed theory-based algorithm; in contrast, miRTarBase was used for collecting experimentally validated target genes. In this experiment, we focused on top 5 high DNS of rmiRNAs, namely, rmiR-1268, rmiR-3656, rmiR-4466, rmiR-6087, and rmiR-6724, and these rmiRNAs were located at separated regions of the rDNA-repeating unit, such as 5′ ETS, 28S rRNA, 3′ ETS, and NTS. Top 10 targets of top 5 DNS rmiRNAs (total 50 targets) were extracted from both TargetScan and miRTarBase (Supplemental Table  2); subsequently, their classification in GO biological process (BP), molecular function (MF), and cellular component (CC) were performed and their results were listed through GeneCodis3 web tool (Figures 2(a) and 2(b)). Intriguingly, three gene ontology (GO) terms, namely, nucleus (CC), protein binding (MF), and nucleotide binding (MF), were commonly ranked on top 3 place between TargetScan and miRTarBase. Moreover, almost all their biofunctions were commonly related to gene regulation such as transcription and nucleotide binding although the greater parts of the GO analysis results were different in detail. Contributions of total 50 targets in biological pathway were also analyzed using GeneCodis3 with Kyoto Encyclopedia of Genes and Genomes (KEGG) pathway option. However, no pathway was presented in both cases of predicted target (TargetScan) and validated targets (miRTarBase). Therefore, to investigate in larger scale, we extracted all predicted targets in TargetScan having more than 0.1 cumulative weighted context++ score. KEGG pathway analysis of these targets was conducted and various biological pathways were successfully indicated. The results showed that the majority of putative targets were related to cancer or cancer-related pathways such as MAPK signaling and ERBB signaling (Figure 2(c)). This suggests that rmiRNAs have an inclination to target cancer-related genes and might have some important roles in anticancer or antistress pathways.

### 3.5. Prediction of rmiRNA Targets in Alu

Numerous Alu element-containing RNAs exist in the nucleolus and participate in the synthesis of rRNAs. Therefore, to seek RNA-RNA relationship between nucleolar function and rmiRNA, target sites of rmiRNAs inside Alu sequence were searched. Six branched members of Alu family, namely, Alu consensus, AluJo, AluSz, AluSc, AluSp, and AluY, were processed using RNA22 tool and then 5, 8, 7, 5, 4, and 3 putative rmiRNA target sites were detected, respectively (Supplemental Table  3). Several rmiRNA target sites were conserved among these Alu sequences. Nine of 17 rmiRNAs have potent target sites on Alu sequences and 3 of 5 high DNS rmiRNAs such as miR-4466, miR-6087, and miR-6724-5p were included.

## 4. Discussion

Ribosomes, large ribonucleoprotein complexes composed of various RPs and rRNAs, are a molecular machine that translate mRNAs into proteins and exists in all living cells [78, 79]. Proper function of the ribosome is essential for normal cell activities; therefore, modification and assembling of RPs and rRNAs are strictly and intricately regulated in the ribosome construction process [78–80]. Ribosomal dysfunction is associated with various diseases represented by ribosomopathies such as DBA, TCS, and SDS and is caused by mutation in ribosome-related genes, ribosomal haploinsufficiency, cellular stresses from chemical or infection, and so forth [1–3]. Several ribosomopathies increase the risk of carcinogenesis and cancer cells often have abnormality in the ribosome function due to mutation in RP and ribosome-related processor genes that cause ribosomopathies [4].

The synthesis of the ribosome itself largely contributes to malignant cell proliferation [81–83]. Ribosomal biogenesis is generally upregulated in the G1 phase dividing cells because enhanced protein synthesis is required to produce viable daughter cells [84]. Thus, inhibiting ribosomal synthesis causes G1 phase arrest and hinders cell proliferation reversibly in normal cells [85]. The upregulation of ribosomal synthesis is also observed in various cancer cells [82, 83]; therefore, inhibiting ribosomal synthesis has been recognized as a potent and novel anticancer strategy [81, 86–88]. This method particularly showed apoptosis-inducing effects in various malignant cells that synthesized ribosomes at a high rate and exhibited sufficient efficacy with only 3 h transient treatments. This inhibition was accomplished by deleting ribosomal protein genes or by cell treatments with actinomycin D, doxorubicin, 5-fluorouracil, and CX-5461, which have significant tumor suppressing effects [85, 88, 89]. However, this antitumor effect of inhibiting ribosomal biogenesis is not dependent on suppressing protein synthesis but on ribosomal biogenesis itself [81]. This anticancer mechanism presumably depends on stabilizing p53 by inhibiting a p53 degrading protein, called Murine Double Minute 2, by competitively combining with a ribosomal protein, which becomes free because it no longer participates in the construction of ribosomes [81, 88]. Additional evidences declaring associations between ribosome and cellular dysfunction have been reported. 28S rRNA has been identified as a novel fusion partner of carcinoma-related genes such as BCL6, BCL11B, IGKV3-20, and COG1 in gastric lymphoma or hematopoietic tumors [90, 91]. Mutation or inhibition of specific genes associated with ribosome construction pathway such as DKC1, AROS, and several snoRNAs have been identified to impair normal cell functions and sometimes cause carcinogenesis in various cell types [92–95]. Angiogenin-mediated rRNA transcription has been revealed to be related to squamous cell carcinoma [96]. Almost all these ribosome-related genes are considered as potent therapeutic targets for ribosomopathies.

Similar to rRNA, tRNA is the second most abundant noncoding RNA and contains many miRNA-like fragments as tRFs. Various tRFs are generated from mature or pre-tRNAs by ordered cleavage processing and they have similar characteristics to miRNAs, such as evolutionary conservation, target RNA recognition, translational regulation, circulation, and interaction with AGO proteins [57, 59–63, 97, 98]. In addition, miRNAs derived from pre-tRNA or tRNA–miRNA encoded by tRNA genes have been reported [59, 99]. Therefore, it is appropriate that miRNAs or miRNA-like functional fragments are also generated from rRNA gene-related regions.

As a sequel to computational analysis, we found that 17 pre-miRNA-like arrays, which contained identical sequences to known human mature miRNAs, were located in the rRNA gene coding region; therefore, we called them rDNA-hosted pre-miRNA analogs (rmiRNA). We also performed supplemental examination with mouse miRNAs and rDNA and found several sequences which are identical to miR-696, miR-712-5p, miR-712-3p, miR-714, miR-466i-5p, miR-5099, and miR-6538. Of these miRNAs, miR-696, miR-712, and miR-714 have already been reported as rRNA-locating ones [47]. According to Son et al., mmu-miR-712 is located at ITS2 of mouse rRNA gene (Rn45s) and hsa-miR-663 is located at ITS1 region of human rRNA gene (RNA45S) [48]. In our research, miR-712 was likewise found in ITS2 of Rn45s; however, hsa-miR-663 was not found in ITS1 but 5′ ETS of RNA45S. This discrepancy may be caused by the differences in detection tool or the version of RNA45S sequence data. For instance, they used MirEval web tool for sequence analysis and their study probably may be based on the older version of rRNA sequence data.

We also discovered that almost all of these rmiRNAs formed stem-loop structures and were located in RNA45S rather than the NTS (Figure 1, Supplemental Figure  2, and Table 2). Putative pre-rmiRNAs which have very similar sequences with their original miRNAs also showed similarity to their original miRNAs in the thermodynamic stability of stem-loop structures (Supplemental Figure  4A). Moreover, putative pre-miRNAs which have moderate substitutions in their sequences except the guide strands likewise showed similar stabilities and some showed more stable stem-loop structures than their original pre-miRNAs. This indicates that rmiRNAs have adequate potential stability to form stem-loop structures regarded as pre-miRNA. These results suggest that a large number of rmiRNAs are continually transcribed because rRNA is the most abundant noncoding RNA in eukaryotic cells. rDNA is transcribed so frequently that the rDNA region on the genome forms multicopies in the nucleolus [100]. Furthermore, rmiRNA may be noncanonically generated from rRNA in the cytoplasm because cytoplasmic RNA is a miRNA source [55].

All pooled miRNAs, such as rmiRNAs and/or tRFs, are important for immunoreactivity, transcriptional regulation, gene mobility, and cytoplasmic memory [17]. Sharma et al. indicated that paternal diet alters RNA information, such as population and composition of tRFs, in spermatozoa and influences progeny phenotype [101]. Likewise, although it was not confirmed in this study whether rDNA-hosted miRNAs would work* in vivo* with known identical miRNAs, rmiRNAs may function as resident miRNAs and participate in determining genotype and memorization. The number of stress-sensitive miRNAs is insufficient to exert an immediate response to cell damage if these miRNAs are generated only by transcription from DNA. Therefore, rapid generation of miRNAs from ready-made RNAs, such as rRNA and tRNA, should be considered. In addition, rmiRNAs and their identical miRNAs may work together because homologous miRNAs at different loci function together [102].

According to previous reports, rRNA-contained miRNAs such as miR-663, miR-1275, miR-3648, miR-3656, miR-3687, miR-4417, and miR-4516 are associated with tumor suppression, carcinomas, neuronal differentiation, breast cancer, breast cancer/neuronal differentiation, breast cancer, and regulation of signal transducer and activator of transcription 3, respectively [103–108]. However, the functions of residual rRNA-hosted miRNAs remain unclear. This motivated us to predict the targets of rmiRNAs and a number of putative and validated target genes which were associated with cancer-related pathways were found (Figure 2(c)). Therefore, RNA-RNA and/or RNA-protein interactions may participate in cancer-related functions. This indicates that rmiRNAs, in addition to ribosome-associated proteins and snoRNAs, might also be implicated in cell dysregulation and dysfunctions linked to ribosomopathies in multiple steps such as transcription, posttranscription, and biofunction.

In our previous study, the DNS was positively correlated with the strength of miRNA/miRNA synergies [72, 109]. As these rmiRNAs commonly have high DNS values, rmiRNA-derived miRNAs may also function as an activity booster of other miRNAs (Supplemental Figure  2). This characteristic would be effective for quick responses to cell emergencies that are not severe enough to cause changes in intracellular RNA composition [10, 16, 110, 111]. In this study, most of the top 10 rmiRNA targets were predicted to play roles in gene regulation and participate in cancer-related pathways (Figure 2(c)). This finding indicates that the rRNA copy number and expression level may be directly associated with rmiRNA generation and regulation of the biological reactions to cell stress leading to carcinogenesis.

Given the known mechanisms of pre-rRNA processing and that of miRNA generation from rRNA, maturation of rmiRNA occurs as follows: (1) rRNA genes containing rmiRNAs are transcribed as RNA45S by Pol I [7, 9]; (2a) the RNA45S ITS and ETS are degraded by XRN1 or other nucleases after the rRNA matures, and pre-rmiRNAs located in these regions are generated simultaneously [7, 9, 48]; (2b) pre-rmiRNAs located on 18S rRNA are biologically generated upon the degradation of mature rRNAs in the ribosome or degradation of pre-rRNA in response to stress [110–112]. (3) Drosha, Dicer, or its related proteins and enzymes process pre-rmiRNAs into mature rmiRNAs. The last step in which Drosha and Dicer participate in rRNA processing has been observed in several studies. RNase III enzymes including Drosha and Dicer have a miRNA-independent role in RNA processing, because the depletion of Dicer or Drosha impairs rRNA processing but does not affect the exonuclease activities required for rRNA processing [113, 114]. Fukuda et al. revealed that the DEAD-box RNA helicase p68 (Ddx5) and p72 (Ddx17), which are subunits of the Drosha complex, are required for pre-rRNA and pri-miRNA processing. Woolnough et al. reported that the human Ago2 protein binds rRNA and interferes with the transcription of nascent human rRNA via binding with Pol III and the transcription factor III complex on the gene [66, 115]. These data indicate that rRNA processing is closely related to the miRNA processing enzymes and its related proteins. In contrast, Chak et al. reported that the generation of miR-10404 and endo-siRNA from the rRNA gene is unaffected by mutations in Drosha, Pasha, Ago2, or Dcr-2 but by Dcr-1 in* Drosophila* [47]. Son et al. demonstrated that the generation of pre-miR-712 is dependent on XRN1 but independent of Drosha and DGCR8 in mice [48]. Pre-miR-712 processing is a mirtron-like, but it remains unknown whether Drosha and Dicer contribute to generating rRNA-derived miRNA because the details of the roles of Drosha, Dicer, and related proteins in rRNA processing are unknown. However, these findings suggest that rRNA-derived miRNAs could be generated in both Drosha-dependent and Drosha-independent pathways.

The number of repeated rDNA arrays is strongly associated with cell senescence, gene integrity, and ribosomal function, although the majority of rDNA is inactive [15–17]. Moreover, rDNA cluster size differs among species and individuals and even in individual cells when the cells are responding to DNA damage or when the rRNA repeat number is being amplified [15–17, 116]. As these differences are inherited, it is certain that rRNA and rRNA-hosted miRNAs participate in cell identity [117]. The ETS and ITS regions are not highly conserved as compared to 18S, 5.8S, and 28S RNA. All three previously reported ITS- or ETS-derived miRNAs, such as miR-663, miR-712, and miR-10404, are human-, mouse-, and fly-specific miRNAs, respectively, and they are well conserved intraspecifically [47, 48], suggesting that variations in rmiRNAs and rDNA copy number contribute to evolution, particularly the inheritance of acquired characteristics.

Nucleolus, where rRNA is transcribed and processed, is the largest structure in the nucleus formed at rRNA coding regions on chromosomes and composed of diverse specific proteins and RNAs [118]. It has been revealed that some miRNAs exist and function in nucleolus. For instance, miR-206, a highly expressed miRNA in skeletal muscle, and several other miRNAs are detected in the nucleolus as well as in the cytoplasm with* in situ* hybridization [119]. Subsequently, it has been shown by deep sequencing that a set of miRNAs present in the nucleus rather than in the cytoplasm and some of them tend to accumulate at the nucleolus [120]. RNA interference (RNAi) factors such as AGO protein, Dicer, and TRBP are also found in the cell nuclei, suggesting that miRNA machinery is active even in the nucleolus [121]. Moreover, it has recently been reported that Alu element-containing Pol II transcripts (aluRNA) are abundant in nucleolus [67]. Alu element is the most abundant SINE family that comprises about 10% of the genome and exists in both noncoding and coding region including introns and 3′ UTR of mRNA transcripts [122, 123]. There are growing evidences that a portion of mRNAs have Alu-derived sequence in their 3′ UTR which can be targeted by a set of miRNAs [124–126]. Since it has been reported that the transcriptional rate of Alu is upregulated upon cellular stress and strongly influences the nucleolar size and pre-rRNA transcript rate [67, 127, 128], we supposed that aluRNAs might also be regulated by miRNAs. In our investigation, several miRNAs were detected from rRNA sequence as rmiRNA, and half of these rmiRNAs have potential target sites in Alu family sequences (Supplemental Table  3). Although it was not confirmed in this study whether rmiRNAs really regulate aluRNAs, at least, the possibility that rmiRNAs might interact with aluRNA in the nucleolus and contribute to the regulation of ribosomal function and composition upon cellular stress as a ribosomal feedback machinery was implied.

It was technically difficult to distinguish the origins of the sources using ready-made technologies, because the mature rmiRNA sequences, such as rmiR-663a/b and rmiR-1268a/b, were identical between the rDNA and non-rDNA genes. Therefore, in this study, we performed* in silico* analyses to by-pass this problem. No rmiRNA was detected from the 5S rRNA gene but the AGO2 protein binds to 5S rRNA [115], and AGO2 has Slicer activity [129], suggesting that various rRNA-derived specific miRNAs with different mature sequences to annotated miRNAs, that is, novel miRNAs, may be generated from the rRNA coding region. Furthermore, mature rmiRNA may have been generated in another form, such as loop miRNAs [130]. Numerous undefined RNA fragments derived from well-known RNAs or other noncoding RNAs might unveil the RNA wave enigma and implication of tumorigenesis. Therefore, additional laboratory and clinical investigations are required for discovery of the nascent human miRNAs and for decipherment of precise interaction among miRNAs, noncoding RNAs, and human cancer.

## 5. Conclusion

Seventeen rDNA-hosted miRNA analogs (rmiRNAs) were found in rRNA coding region by* in silico* analyses. These rmiRNAs might be generated from rRNA upon construction or degradation of ribosomes. The majority of predicted targets of rmiRNAs were stress- or cancer-related genes and it was indicated that rmiRNAs could also target AluRNA in nucleolus, suggesting that rmiRNAs may regulate ribosomal function at multiple levels adopting to cellular stress. While rmiRNAs showed significantly high DNS values compared to those of normal miRNAs and tRFs, rmiRNAs may efficiently boost bioactivities of other miRNAs to attenuate cell stress and tumorigenesis as a quantum memory device and a member of the resident miRNA genes. Altogether, rmiRNAs would be implicated in human ribosomopathy. In future, rmiRNA mimics or anti-rmiRNA agents may be developed to cancer therapy and there is some possibility that rmiRNAs in serum could be applied for prognosis and/or diagnosis of ribosomopathy.

## Supplementary Material

Supplemental Figure 1. Sequences and secondary structures of guide-only rmiRNAs. Supplemental Figure 2. Sequences and secondary structures of reversed rmiRNAs. Supplemental Figure 3. DNS distribution of rmiRNA, tRF, and all miRNAs. Supplemental Figure 4. Free energies of secondary structures of pre-miRNAs and their original pre-miRNAs. Supplemental Table 1. Differences between two sample rRNA gene sequence. Supplemental Table 2. TOP 10 predicted and validated targets of rmiRNAs. Supplemental Table 3. Predicted interactions between Alu family and rmiRNA.

## Figures and Tables

**Table 1:** Detected mature miRNAs from rRNA gene and adjacent region.

miR name	Mature sequence	Region	Location
miR-663a	AGGCGGGGCGCCGCGGGACCGC	5′ ETS	2049–2071
miR-663b	GGUGGCCCGGCCGUGCCUGAGG	5′ ETS	2113–2135
miR-1268a	CGGGCGUGGUGGUGGGGG	3′ ETS	13102–13119
miR-1268b	CGGGCGUGGUGGUGGGGGUG	3′ ETS	13102–13121
miR-1275	GUGGGGGAGAGGCUGUC	(NTS)	42294–42310
miR-3648	AGCCGCGGGGAUCGCCGAGGG	5′ ETS	2513–2533
miR-3656	GGCGGGUGCGGGGGUGG	28S	8524–8540
miR-3687	CCCGGACAGGCGUUCGUGCGACGU	(5′ ETS)	2888–2911
miR-4417	GGUGGGCUUCCCGGAGGG	5′ ETS	2412–2429
miR-4466	GGGUGCGGGCCGGCGGGG	(5′ ETS)	631–648
miR-4488	AGGGGGCGGGCUCCGGCG	28S	8510–8527
miR-4492	GGGGCUGGGCGCGCGCC	28S	10851–10867
miR-4508	GCGGGGCUGGGCGCGCG	28S	10849–10865
miR-4516	GGGAGAAGGGUCGGGGC	28S	11049–11065
miR-4532	CCCCGGGGAGCCCGGCG	28S	11227–11243
miR-6087	UGAGGCGGGGGGGCGAGC	28S	12007–12024
miR-6724	CUGGGCCCGCGGCGGGCGUGGGG	(NTS)	42320–42342

Seventeen sequences homologous to mature human miRNAs were detected from rRNA gene coding region. Note that miR-1268a and miR-1268b were found in only RNA45S and miR-1275, miR-3687, miR-4466, and miR-6724 were found in only rDNA-repeating unit. This data might be caused by differences in base alignment between two rRNA sequence data.

**Table 2:** A list of all determined pre-rmiRNA sequence and its location.

rmiR name	Sequence of pre-rmiRNAs	Region	Location
rmiR-663a	CCGUCCGGCGUCCCAGGCGGGGCGCCGCGGGACCGCCCUCGUGUCUGUGGCGGUGGGAUCCCGCGGCCGUGUUUUCCUGGUGGCCCGGCCGUG	ETS	2028–2119
rmiR-663b	GGGGCCGAGGGCCGUCCGGCGUCCCAGGCGGGGCGCCGCGGGACCGCCCUCGUGUCUGUGGCGGUGGGAUCCCGCGGCCGUGUUUUCCUGGUGGCCCGGCCGUGCCUGAGGUUUC	ETS	2025–2140
rmiR-1268a	CUUCCUCCCUCCCGGCCUCUCCCGCCGACCGCGGGCGUGGUGGUGGGGGU	3′ ETS	13071–13123
rmiR-1268b	CCGCGGGCGUGGUGGUGGGGGUGUGGGGGGGAGGGCGCGCGACCCCGGUCGGCGCGCCCCGCUUC	3′ ETS	13099–13163
rmiR-1275	AGCCCGGCUGGCCCGGUGGCGCCAGAGCUGUGGCCGGUCGCUUGUGAGUCACAGCUCUGGCGUGCAGGUUUAUGUGGGGGAGAGGCUGUCGCU	(NTS)	42221–42313
rmiR-3648	CGCGACUGCGGCGGCGGUGGUGGGGGCAGCCGCGGGGAUCGCCGAGGGCCGGUCGGCCGCCCCGGGUGCCGCGCGGUGCCGCCGGCGGCGGUGAGGCCCCGCGCGUGUGUCCCGGCCGCGGUCGGCCGCGCUCGAGGGGUCCCCGUGGCGUCCCCUUCCCCGCCGGCCGCCUUUCUCGCG	ETS	2486–2665
rmiR-3656	CUCCCUUCCCCCGCCGCCCCUCCUCCUCCUCCCCGGAGGGGGCGGGCUCCGGCGGGUGCGGGGGUGGGC	28S	8474–8542
rmiR-3687	CGCGCGUGCGCCCGAGCGCGGCCCGGUGGUCCCUCCCGGACAGGCGUUCGUGCGACGUGUG	(ETS)	2854–2914
rmiR-3687^*∗*^	CGCGCGUGCGCCCGAGCGCGGCCCGGUGGUCCCUGCCGGACAGGCGUUCGUGCGACGUGUG	ETS	2857–2917
rmiR-4417	GCGUGGGGCCCGGUGGGCUUCCCGGAGGGUUCCGGGGGUCGGCCUGCGGCGCGU	ETS	2400–2454
rmiR-4466	UCGCGGGUGCGGGCCGGCGGGGUCCUCUGACGCGGCAGACAGCCCUGCCUGUCG	(ETS)	627–680
rmiR-4488	CCGCCCUCCCUUCCCCCGCCGCCCCUCCUCCUCCUCCCCGGAGGGGGCGGGCUCCGGCGGGUGCGGGGGUGGGCGG	28S	8468–8544
rmiR-4492	GGGGCGCGAAGCGGGGCUGGGCGCGCGCCGCGGCUGGACGAGGCGCCGCCGCCCCCCCCACGCCCGGGGCACCCCCCUCGCGGCCC	28S	10838–10924
rmiR-4508	GGCGCGAAGCGGGGCUGGGCGCGCGCCGCGGCUGGACGAGGCGCCGCCGCCCCCCCCACGCCCGGGGCAC	28S	10841–10910
rmiR-4516	CCGUCCUCCCCCCUCCCCGGGGGAGCGCCGCGUGGGGGCGGCGGCGGGGGGAGAAGGGUCGGGGCGG	28S	11001–11067
rmiR-4532	GACGCGAGCCGGGCCCUUCCCGUGGAUCGCCCCAGCUGCGGCGGGCGUCGCGGCCGCCCCCGGGGAGCCCGGCGGGCGCCGGCGC	28S	11169–11254
rmiR-6087	GGUGAGGCGGGGGGGCGAGCCCCGAGGGGCUCUCGCUUCUGGCGCCAAG	28S	12005–12052
rmiR-6724	CGCUGCGCUUCUGGGCCCGCGGCGGGCGUGGGGCUGCCCGGGCCGGUCGACCAGCGCGCCGUAGCUCCCGAGGCCCGAGCCGCGACCCGGCG	(NTS)	42310–42401

Note that some of them are overlapping each other. ^*∗*^rmiR-3687 indicates the sequence identical with pre-miR-3687 except for a point mutation in the guide sequence (see the lower part of Figure 1(a)).
