# Supplementary material for: Human Ribosomal RNA-Derived Resident MicroRNAs as the Transmitter of Information upon the Cytoplasmic Cancer Stress
Source: Biomed Res Int. 2016 Jul 19;2016:7562085. doi: 10.1155/2016/7562085 (PMC4969525; doi:10.1155/2016/7562085)

	RNA45S (NR_046235.1)			rDNA-repeating unit (U13369.1)		
	Start	End	Length	Start	End	Length
5’ ETS	1	3654	3654	1	3656	3656
18S	3655	5523	1869	3657	5527	1871
ITS1	5524	6601	1078	5528	6623	1096
5.8S	6602	6757	156	6624	6779	156
ITS2	6758	7924	1167	6780	7934	1155
28S	7925	12994	5070	7935	12969	5035
3’ ETS	12995	13357	363	12970	13314	345
NTS	-	-	-	13315	42999	29685
Total length	**13357**			**42999**		

Supplemental Table 1. Comparison between two rRNA gene sequence data.

Two rRNA gene data showed differences in its length and moderate amount of insertion (or deletion) and substitutions.

rmiR	miR-1268	miR-6724	miR-3656	miR-4466	miR-6087
**DNS**	196	196	169	169	169
**TOP10 Target Genes (TargetScan)**	MUC2	AL627309.1	CLCN6	NNAT	CXXC11
	SLC8A2	BOK	ADAMTS17	ICAM3	RHOG
	EHD2	PPP2R4	THRA	CAMK2B	TAPBP
	LGI3	HMGA1	DBNDD1	FBXL16	FGFRL1
	HOGA1	SUMF2	BARX1	GRAMD1A	OTP
	AL627309.1	CD6	RANBP3	LTBP4	CTF1
	RASD2	CHTF8	RAB20	MT-ND5	PRRT1
	EGR4	FGFBP2	C19orf77	PHLDA1	C15orf37
	ADAMTS4	ANG	TOR4A	CDKN2A	ZNF726
	SCGB1A1	GNG12	BLOC1S1	G6PC3	KHDC3L
**TOP10 Target Genes (miRTarBase)**	QSOX2	MTFMT	MRPL12	NAGK	PSMG1
	AIP	SUMF2	LSP1	NFX1	HAVCR1
	WWP2	ADAMTS13	MNT	F2R	TANGO2
	BARX1	VPS37B	PRDM2	DDA1	ZNF394
	SGPL1	SP2	ZNF770	REXO1	KBTBD12
	POLR2I	PPP6R1	CECR1	KMT2D	CECR1
	FASN	COPS7B	TBC1D19	AACS	CCDC125
	SOX12	ISL2	NUP43	MICAL2	MYADM
	CAMK2G	SOX12	ATG9A	GGA2	NMNAT1
	MAPK1	VPS51	RASL10B	MINOS1	HECTD3

Supplemental Table 2. Top 10 target genes of rmiRNAs.

Predicted targets were collected from TargetScan and experimentally validated targets were collected from miRTarBase.

	miRNA name	sequences	folding energy (-Kcal/mol)	leftmost position of predicted target site	p-value
**Alu**	hsa-miR-4492	GGTGAAACCCCGTCTC	-15.3	99	0.0207
	hsa-miR-663b	GCAGTGAGCCGAGATCGCGCCACT	-12.5	220	0.0774
	hsa-miR-4466	TCGCGCCACTGCACTC	-20.9	234	0.0774
	hsa-miR-4492	AGAGCGAGACTCCGTCTC	-12.3	263	0.111
	hsa-miR-6087	GAGCGAGACTCCGTCTCA	-19.2	264	0.111
**AluJo**	hsa-miR-4488	AGCGAGACCCCGTCTCTA	-17.9	99	0.00592
	hsa-miR-4492	ATAGCGAGACCCCGTCTC	-15.2	101	0.00592
	hsa-miR-6087	GCGAGACCCCGTCTCT	-20.3	102	0.00592
	hsa-miR-6724-5p	GGTGGCGCGCGCCTGTAGTCCCAG	-25.4	146	0.000525
	hsa-miR-663b	AGTGAGCTATGATCGCGCCACT	-15.3	224	0.0774
	hsa-miR-4466	TCGCGCCACTGCACTC	-20.9	236	0.0774
	hsa-miR-663a	GCGACAGAGCGAGACCCTGTCT	-20.8	260	0.29
	hsa-miR-6087	GAGCGAGACCCTGTCTCA	-20	266	0.29
**AluSc**	hsa-miR-6724-5p	GGTGGCGCGCGCCTGTAGTCCCAG	-25.4	144	0.000525
	hsa-miR-3648	TACTCGGGAGGCTGAGGCA	-13.8	169	0.363
	hsa-miR-4466	TCGCGCCACTGCACTC	-20.9	234	0.0616
	hsa-miR-4492	ACTGCACTCCAGCCTG	-16.4	241	0.0616
	hsa-miR-4508	TGCACTCCAGCCTGGC	-21.1	243	0.0616
**AluSp**	hsa-miR-3648	TACTCGGGAGGCTGAGGCA	-13.8	171	0.363
	hsa-miR-4466	TCGCGCCATTGCACTC	-16.6	236	0.242
	hsa-miR-4492	ATTGCACTCCAGCCTG	-16.2	243	0.242
	hsa-miR-4508	TGCACTCCAGCCTGGG	-17.7	245	0.242
**AluSz**	hsa-miR-663a	GGAGTTCGAGACCAGCCTGGCCA	-16.5	74	0.0207
	hsa-miR-4492	GGTGAAACCCCGTCTC	-15.3	76	0.0207
	hsa-miR-4508	AGTTCGAGACCAGCCTGGC	-19.9	101	0.0207
	hsa-miR-663a	GCGCGCCTGTAATCCCAGCTA	-17.9	152	0.0382
	hsa-miR-3648	TACTCGGGAGGCTGAGGCA	-13.8	171	0.363
	hsa-miR-663b	GCAGTGAGCCGAGATCGCGCCACT	-12.5	222	0.0774
	hsa-miR-4466	TCGCGCCACTGCACTC	-20.9	236	0.0774

**AluY**	hsa-miR-4492	GGTGAAACCCCGTCTC	-15.3	99	0.00566
	hsa-miR-663b	GCAGTGAGCCGAGATCGCGCCACT	-12.5	221	0.0774
	hsa-miR-4466	TCGCGCCACTGCACTC	-20.9	235	0.0774

Supplemental Table 3. A list of predicted target sites of rmiRNA in Alu family. Sequence of Alu family is well conserved but number of targeting rmiRNAs and p-value are moderately differed among 6 Alu family.

Supplemental Figure 1

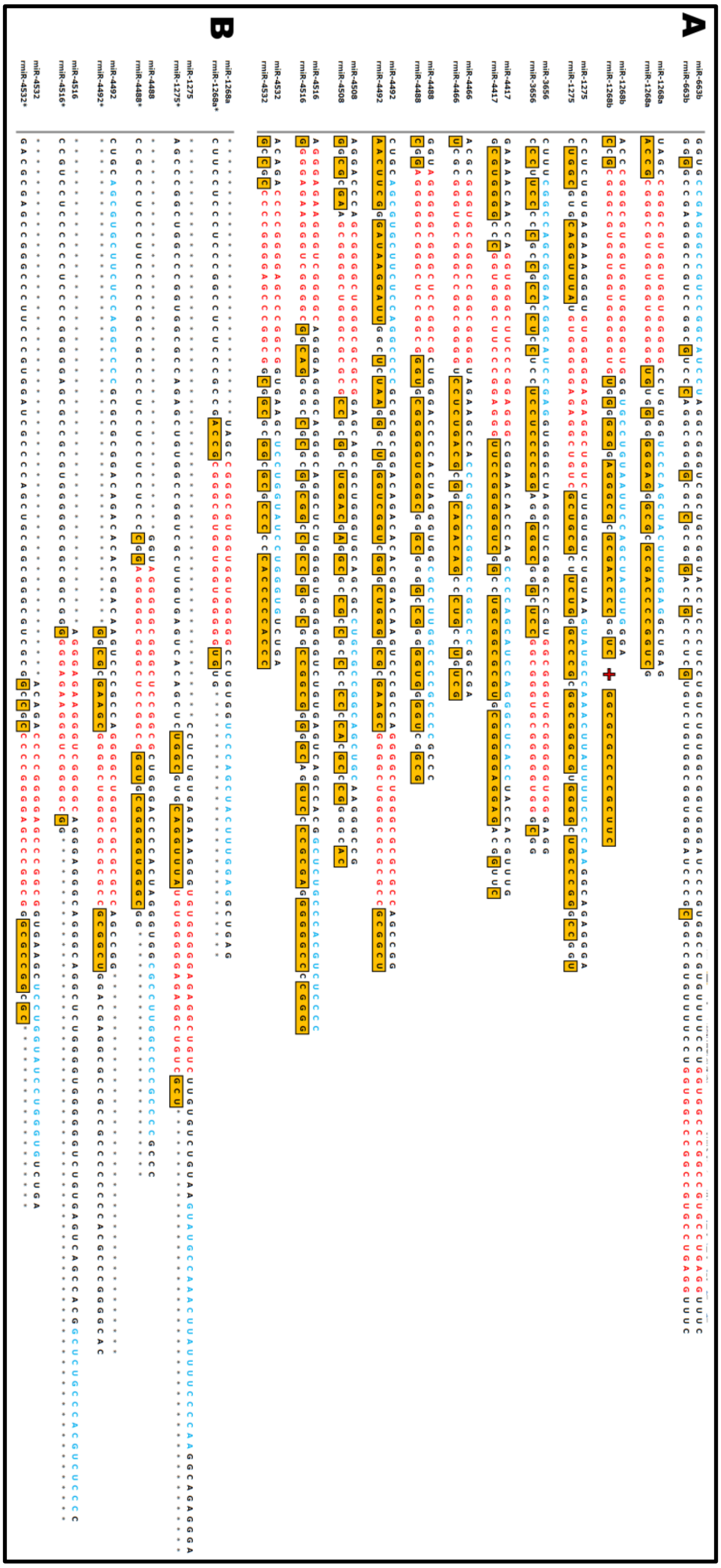

Supplemental Figure 2

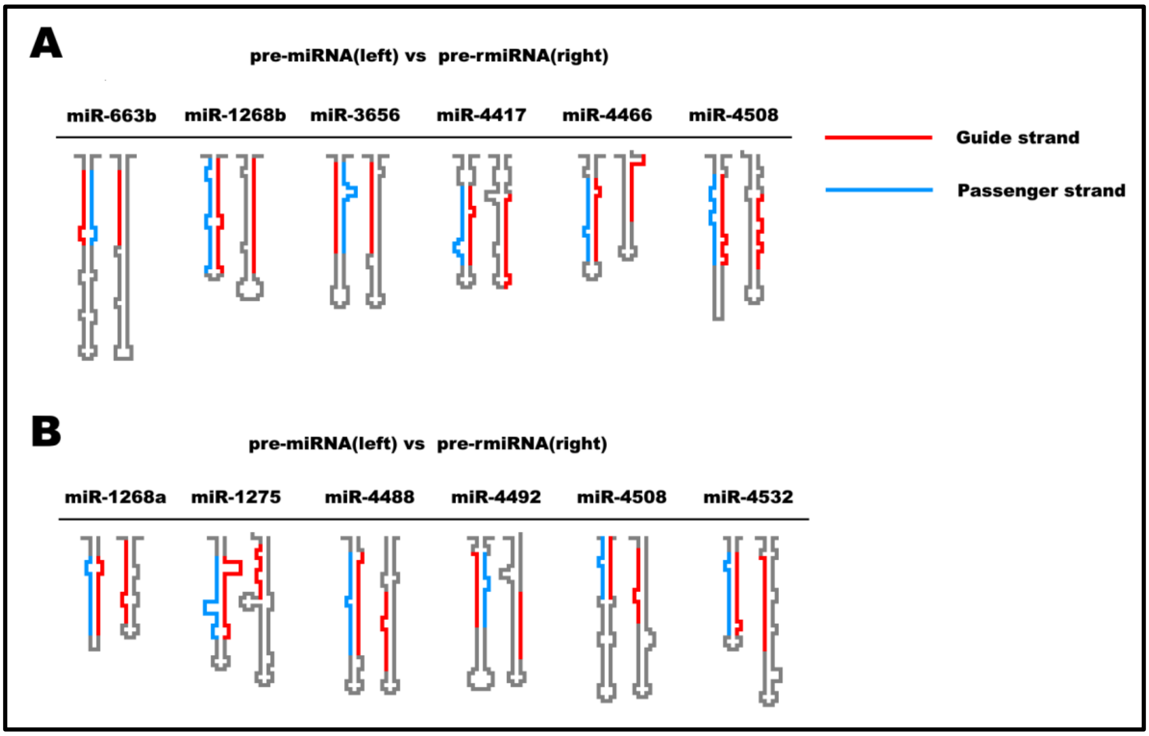

Supplemental Figure 3

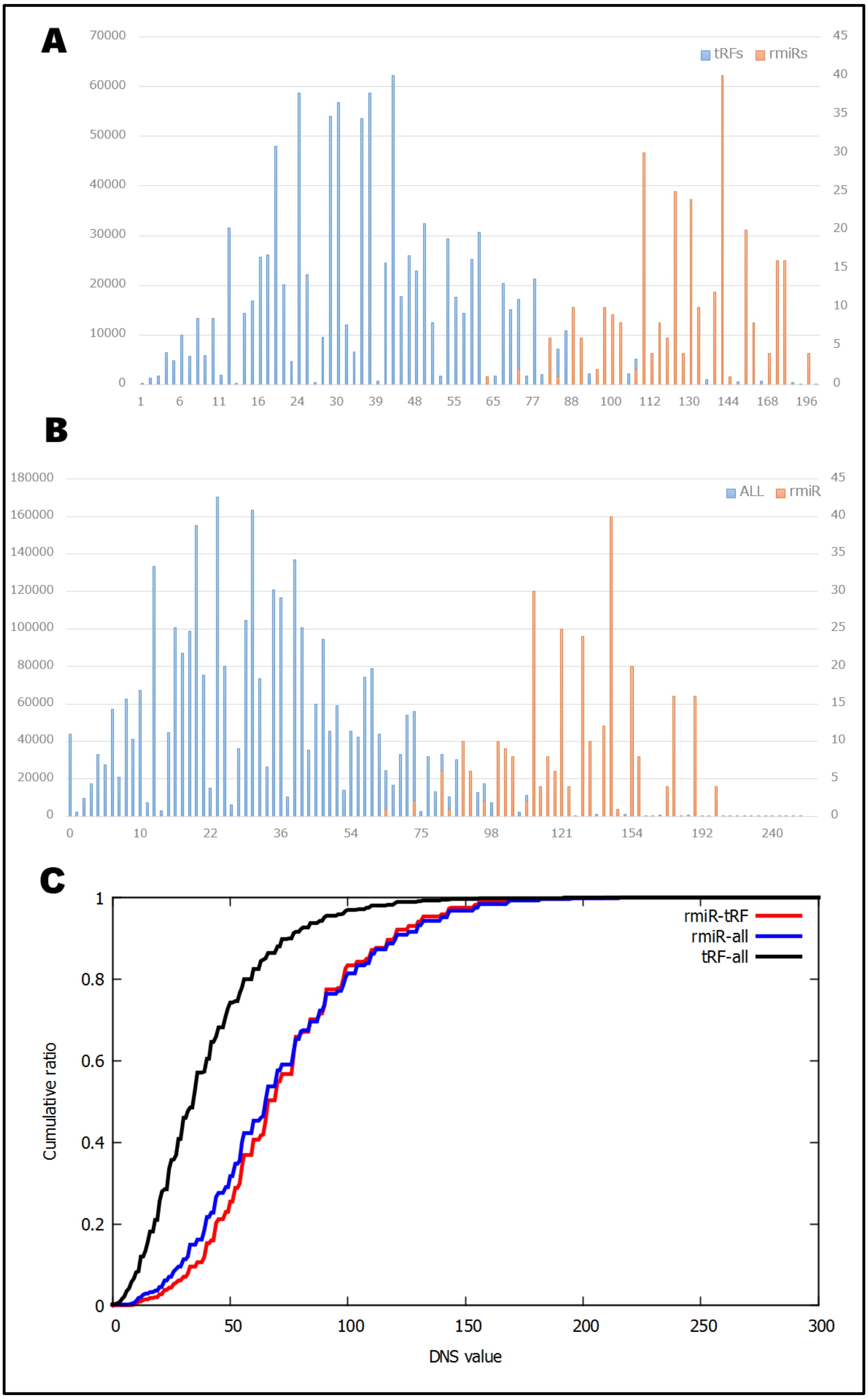

Supplemental Figure 4

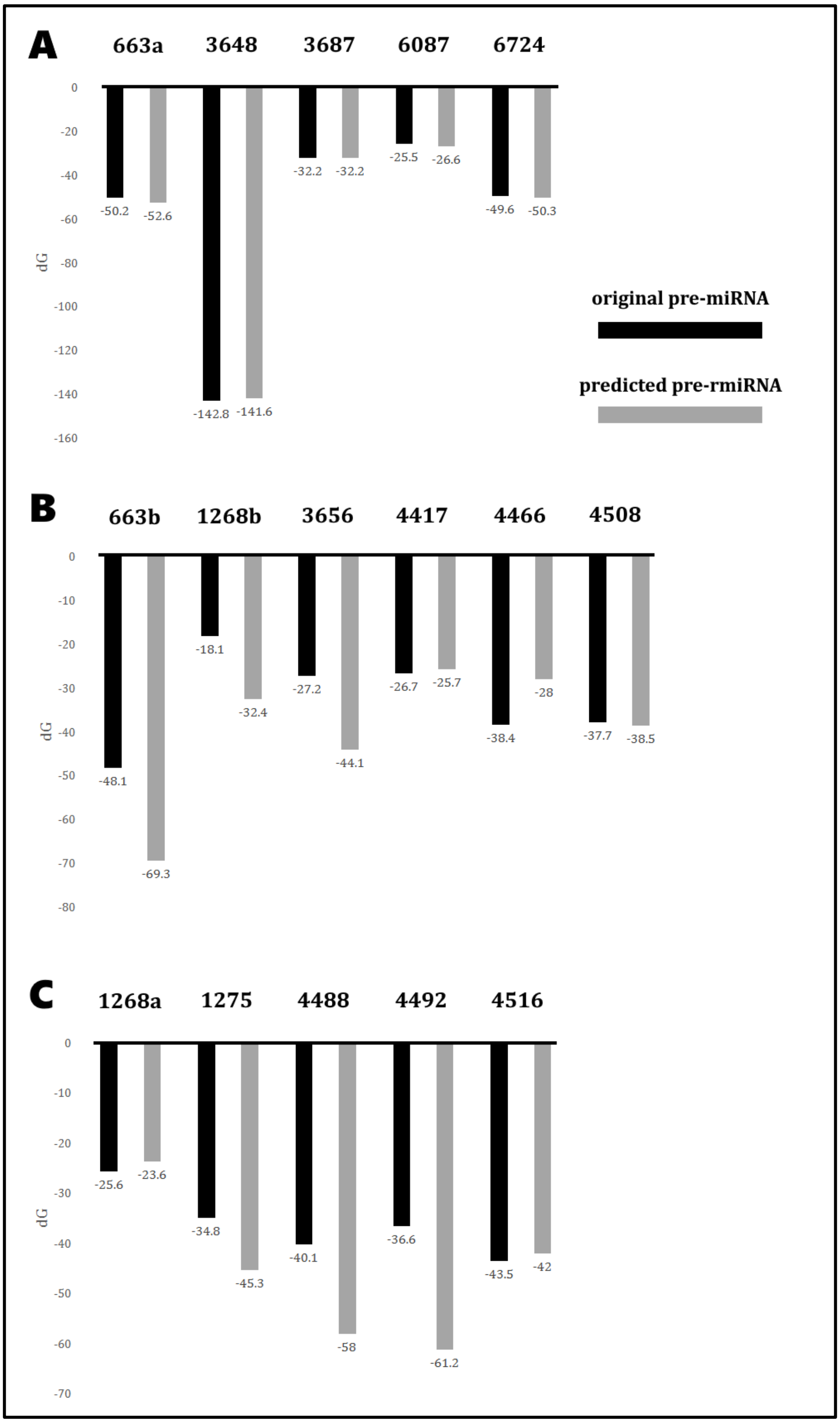

Supplement: Supplementary file 1 — Supplemental Figure 1. Sequences and secondary structures of guide-only rmiRNAs. Supplemental Figure 2. Sequences and secondary structures of reversed rmiRNAs. Supplemental Figure 3. DNS distribution of rmiRNA, tRF, and all miRNAs. Supplemental Figure 4. Free energies of secondary structures of pre-miRNAs and their original pre-miRNAs. Supplemental Table 1. Differences between two sample rRNA gene sequence. Supplemental Table 2. TOP 10 predicted and validated targets of rmiRNAs. Supplemental Table 3. Predicted interactions between Alu family and rmiRNA. [file 7562085.f1.pdf]
